# Factors Contributing to the Duration of Chemotherapy-Induced Severe Oral Mucositis in Oncopediatric Patients

**DOI:** 10.3390/ijerph15061153

**Published:** 2018-06-01

**Authors:** Lecidamia Cristina Leite Damascena, Nyellisonn Nando Nóbrega de Lucena, Isabella Lima Arrais Ribeiro, Tarciana Liberal Pereira de Araujo, Ricardo Dias de Castro, Paulo Rogério Ferreti Bonan, Eufrásio de Andrade Lima Neto, Luiz Medeiros de Araújo Filho, Ana Maria Gondim Valença

**Affiliations:** 1 Departament of Statistics, Universidade Federal da Paraíba, João Pessoa, Paraíba 5045, Brazil; nyellisonobrega@hotmail.com (N.N.N.d.L.); isabella_arrais@yahoo.com (I.L.A.R.); tarcianalp@gmail.com (T.L.P.d.A.); eufrasio@de.ufpb.br (E.d.A.L.N.); luiz@de.ufpb.br (L.M.d.A.F.); 2 Departament of Clinical and Social Dentistry, Universidade Federal da Paraíba, João Pessoa, Paraíba 50445, Brazil; ricardodiascastro@yahoo.com.br (R.D.d.C.); pbonan@yahoo.com (P.R.F.B.); anamvalenca@gmail.com (A.M.G.V.)

**Keywords:** mucositis, child, cancer

## Abstract

This study analyzes the factors contributing to the duration of severe oral mucositis in oncopediatric patients. A longitudinal study was conducted in the pediatric department of a cancer referral hospital between 2013 and 2017. Seventy-three patients diagnosed with cancer undergoing chemotherapy protocols were analyzed. Oral evaluations were performed using the *Modified Oral Assessment Guide* criteria, and the data were collected from the patients’ records. The Kaplan-Meier method was used to estimate survival curves. Most patients were males (52.1%), of mixed race (“pardo”) (49.3%), with a mean age of 7.56 years (±5.34). There was a predominance of patients diagnosed with solid tumors (52.1%), with no metastasis (86.3%), using natural product chemotherapeutics (56.2%), who had not undergone a bone marrow transplant (97.3%); amputation was observed in 35.6% of patients, while death rates were as high as 8.2%. The survival analysis estimated a mean time of 30.6 days until complete remission of severe oral mucositis. The regression analysis showed that patients over 10 years old had a median mucositis duration 1.4 times greater than those at the age of 10 years or younger. Patients without metastasis had a median mucositis duration 1.7 times greater than those with metastasis (*p*-value ≤ 0.10). Increasing age and the absence of metastasis were conditions that prolonged the duration of severe oral mucositis.

## 1. Introduction

Cancer is a disease in which patient survival is associated with the diagnosis time and disease stage at diagnosis [1]. It is ranked as the second cause of death in Brazil and accounts for 13% of the deaths worldwide [2]. In this context, childhood cancer has a significant number of new cases and accounts for 8% of deaths among children in the age group 1–14 years [2].

Adverse effects of childhood cancer treatment are common in the oral cavity, the most common being mucositis, xerostomia and infections caused by viruses, fungi or bacteria. These conditions cause significant systemic changes, which may contribute to an increased length of hospital stay and compromise the quality of life of these patients [3,4,5]. 

Oral mucositis is characterized by inflammation and the appearance of ulcers in the oral mucosa, which appears swollen, erythematous and friable. These symptoms cause pain, discomfort, dysphagia, a decline in the overall condition and can compromise the entire digestive tract [3,4,6], possibly causing odynophagia, malnutrition, dehydration and changes in mood and sleep [7]. The condition can also interrupt chemotherapy and thus hinder the efficacy of the treatment regimen [8,9]. In this regard, remission of mucositis prevents treatment from being interrupted, increases the likelihood of the effective therapeutic management of the disease and consequently increases the likelihood of the patient being cured. 

Chemotherapy can be aggressive toward the mucosa due to its effects on poorly differentiated cells or on those with a high mitosis rate. The effect of chemotherapy is directly toxic and, in some cases, drugs are secreted by saliva, causing damage to the oral cavity [10]. Furthermore, such drugs may affect other tissues, e.g., bone marrow, causing a reduction in immunity and leading to infections and/or oral bleeding. These occurrences can cause indirect stomatotoxicity [11], resulting in the onset of oral mucositis.

Other factors that affect the onset of mucositis include the dosage, duration of treatment and tumor type [12], in addition to factors such as oral hygiene and conditions intrinsic to the patient. As oral mucositis is a multifactorial event, it is crucial to understand how different clinical and treatment variables can be related not only to its occurrence but also to its development and duration. 

With this regard, not only the onset but also the severity of oral mucositis should be considered. At the early stages of the disease, the affected patient remains capable of eating solid foods. On the other hand, severe oral mucositis has a harmful effect on the whole organism, causing changes in the diet, and contributing to parenteral food intake, dependence and weight loss among other effects [13]. In some cases, it is necessary to interrupt the oncological treatment, which would directly influence the patient’s prognosis and survival [9].

Several studies [10,12,13,14,15] have pointed to oral mucositis as an event that can complicate the clinical condition of pediatric oncological patients. However, the duration of mucositis or how the manifestation period of this condition can negatively affect the treatment of these patient remain to be determined. Therefore, this study aims to analyze whether clinical and treatment-related variables are factors contributing to the duration of severe oral mucositis in oncopediatric patients.

## 2. Materials and Methods

This longitudinal, retrospective and quantitative study evaluated information regarding instituted chemotherapy and test results contained in each patient’s records. 

This study was conducted in the Pediatric Department of Napoleão Laureano Hospital in the city of João Pessoa, Paraíba state (PB), Northeastern Brazil, which is a referral center for the prevention, diagnosis and treatment of cancer for the entire state of Paraíba. It constitutes the main facility of the Napoleão Laureano Foundation, a philanthropic entity operating under the federal, state and city authorities.

The hospital treated 7352 cases of cancer in 2015. In the pediatric sector, the clinic treats approximately 150 cases per month [16]. 

This study included patients undergoing cancer treatment, aged between 0 and 19 years, who agreed to participate in the research and who developed severe oral mucositis during antineoplastic treatment. Patients who declined to participate in the study, had recurrence of malignancy or underwent treatment that did not include chemotherapy as one of its therapeutic modalities were excluded from the sample. A total of 73 patients of both sexes diagnosed with solid or hematological malignancies and treated with chemotherapeutic protocols were included in the final sample.

Data collection was performed weekly, and each patient was evaluated for ten consecutive weeks immediately after the start of chemotherapy treatment. Oral evaluations were performed between April 2013 and July 2015 using the *Modified Oral Assessment Guide* (OAG) criteria proposed by Cheng, Chang and Yuen [17] by a trained and calibrated researcher (kappa 0.82—excellent agreement). In order to diagnose oral mucositis, the examiner—who evaluated the oral cavity during all periods of the study—was calibrated by a gold-standard researcher (Doctor in Stomatology) using the modified Oral Assessment Guide. Calibration took place at the dental office of the Napoleão Laureano Hospital where 20 patients in the age group of 0 to 19 years undergoing cancer treatment only with chemotherapy were evaluated.

The OAG is an easily applied instrument that contains eight items (voice, swallowing, lips, tongue, saliva, oral/palate mucosa, labial mucosa and gingiva), each with a range of 1 to 3, in which 1 indicates normal conditions, 2 represents a finding of moderate changes, and 3 represents severe oral mucositis. In this study, 3 was categorized as the presence of severe oral mucositis, with outcome 1, and OAG stages 1 and 2 were grouped as the absence of severe oral mucositis (outcome 0). Stage 1 indicates normal chewing, swallowing and speaking functions, with normal appearance of the mucosa and saliva. Stage 2 indicates a slight damage to the oral structures and functions without lesions, whereas stage 3 consists of tissue ulceration, with or without bleeding; difficulty in some functions such as swallowing, chewing and/or speaking; and absent saliva. If any of the eight items matched stage 3 requisites, then the patient was diagnosed with severe oral mucositis.

The sociodemographic aspects sex, age, skin color (self-reported) and the clinical aspects, tumor type (hematological or non-hematological), amputation, death, bone marrow transplantation (BMT), metastasis and chemotherapy classes (alkylating, antimetabolites, natural products and miscellaneous), were included as explanatory variables. The dependent variable was the presence of severe oral mucositis. 

Remission of severe oral mucositis was considered to be the failure time and was counted in full days up to occurrence of the event. “Censors” (segment cases that did not present the event of interest) in this study were due to deaths or the absence of remission of mucositis during the study period. 

Initially, a descriptive analysis was performed to show the absolute and percentage distributions of the variables in the study sample. To analyze factors that contributed to the duration of severe oral mucositis in oncopediatric patients, the Kaplan-Meier method was used to estimate survival curves which were compared using the log-rank and Peto tests. A significance level of 10% was adopted.

Parametric tests were used to estimate the survival data more accurately. The exponential, log-normal and Weibull distribution models were applied. Graphical tests were used for the choice of model fit method to be used, followed by administration of the maximum likelihood test. The following results were obtained: exponential *p* = 2.6020 × 10^−6^; Weibull *p* = 1.2255 × 10^−5^; and log-normal *p* = 0.0187. The exponential and Weibull values were below the prescribed p-value of 0.01; thus, the null hypothesis of this model fit method was rejected. However, the log-normal value did not result in hypothesis H0 being rejected and was, therefore, used for modeling survival. 

The final regression model was obtained with log-normal distribution to identify the effects of the independent variables on survival. To evaluate the overall quality of fit, Cox-Snell and standardized ê_i_* residuals were used. Some variables were re-categorized in the inferential analysis: the age variable (agec) was categorized into patients aged less than or equal to 10 years (1) and patients over 10 years (2), the skin color variable (skincolorc) was divided into white and non-white, and the treatment variable (treatmentc) was divided into chemotherapy and other treatment types. Skin color information was self-reported in accordance with the most recent national epidemiological oral health survey carried out in Brazil, the SB Brazil 2010 [18]. 

Database processing and analysis were performed using free R software, version 3.2.4. This software is an open source and allows for vast statistical analysis, database management and graphical analysis. To perform the survival analysis, it was necessary to install the “survival” and “flexsurv” packages to perform the maximum likelihood test. The significance level adopted for the final model fit was 10%. 

## 3. Results

Most patients were males (*n* = 38; 52.1%), with a mean age of 7.56 years (±5.34), and the majority identified as mixed race (“pardo”) (*n* = 36; 49.3%). 

A total of 52.1% (*n* = 38) of patients were diagnosed with non-hematological tumors. The antineoplastic protocols were found to be as follows: use of chemotherapeutic agents alone (*n* = 46; 63.0%); chemotherapy associated with surgery (*n* = 19; 26.0%); a combination of chemotherapy, radiotherapy and surgery (*n* = 5, 6.8%); and chemotherapy associated with radiotherapy (*n* = 3; 41.8%). The classes of drugs used in the treatment regimens were as follows: alkylating agents—21.9% (*n* = 16), antimetabolites—38.4% (*n* = 28), natural products—56.2% (*n* = 41), and miscellaneous—11.0% (*n* = 8). 

Most patients did not experience occurrence of metastasis (*n* = 63; 86.3%), neither underwent bone marrow transplantation (*n* = 71; 97.3%). Amputation was performed in 35.6% (*n* = 26) of cases, and death occurred in 8.2% of patients (*n* = 6). The evaluated variables are shown with their complete distribution in Table 1. 

**Figure 1:**
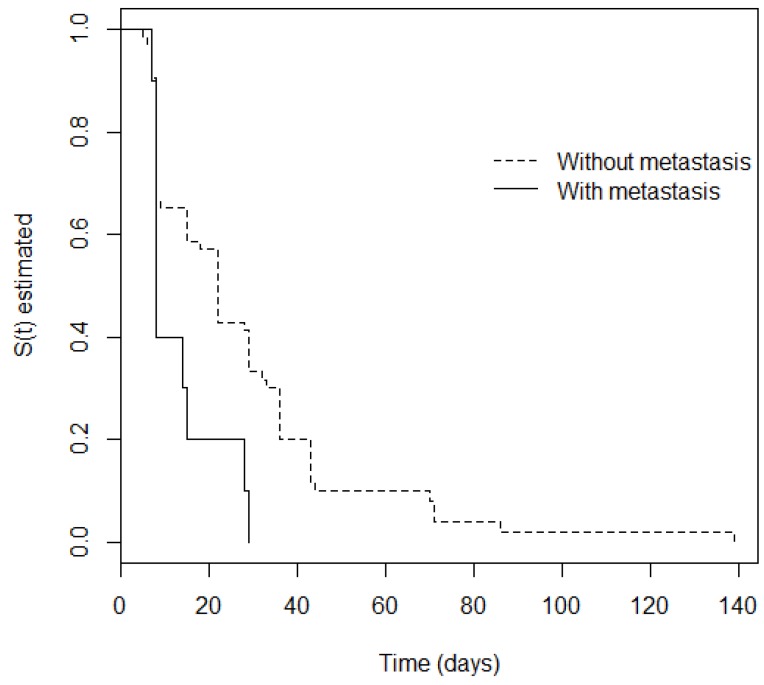
Kaplan-Meier analysis stratified for the metastasis variable of oncopediatric patients with severe oral mucositis in Napoleão Laureano Hospital. João Pessoa, Paraíba State, Brazil (2013–2017).

The non-parametric Kaplan-Meier estimator was initially used and showed an estimated mean time of 30.6 days until remission of mucositis. The log-rank and Peto tests were used to establish whether there was a difference in time for regression of mucositis for each variable. Table 2 shows that only the “Metastasis” variable was considered significant (*p* < 0.10); i.e., there was a difference in mucositis regression time between patients with metastasis and those who did not have it. Figure 1 shows the stratified survival curve for the metastasis variable, showing a significant *p*-value.

When the log-normal model was applied, all variables were initially included, as shown in Table 3. The backward technique was used to remove those that were not significant for the model fit, which resulted in two significant variables when a *p* < 0.10 was considered.

**Figure 2:**
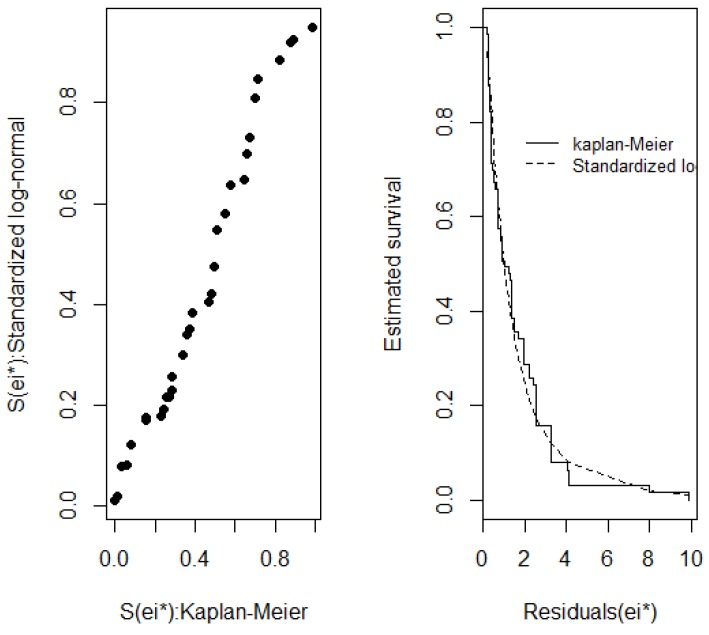
Standardized ê_i_* residuals evaluating the overall final model fit.

**Figure 3:**
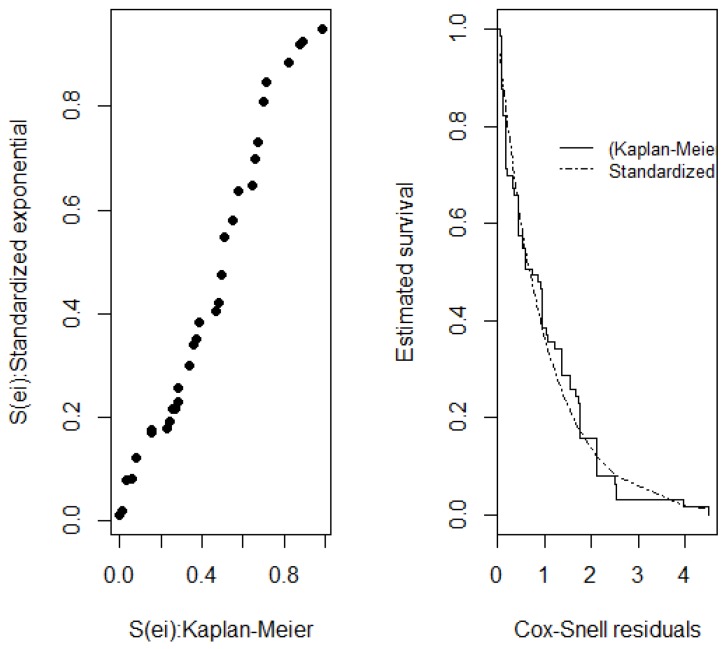
Cox-Snell residuals evaluating the overall final model fit.

The Cox-Snell and standardized ê_i_* residuals, shown in Figure 2 and Figure 3, were used to evaluate overall fit and showed that the log-normal distribution was in line with the Kaplan-Meier survival curve, hence confirming the overall model fit for the data under analysis. The next step was to obtain the ratio of median times for the final model, as described in Table 4. 

The coefficients indicate that patients without metastasis had a median severe oral mucositis duration 1.7 times (1/0.5945205) greater than that of patients who had metastasis. Furthermore, patients over 10 years old had a median severe oral mucositis duration 1.4 times greater than that of patients aged 10 or under.

The estimated mean time of total remission of mucositis was 30.6 days (almost four weeks). It was also verified the rate of Methotrexate (MXT) (therapeutic doses) uses on metastatic and non-metastatic patients and considering patients divided by age strata during first four weeks and-during whole ten weeks of treatment (Table 5). Cleary, non-metastatic and older patients were more exposed to MXT in comparison to other groups. 

## 4. Discussion

The present study identified age and metastasis as factors that affect the duration of severe oral mucositis. These findings are relevant due to their novelty and provide information not previously addressed in the literature. While no studies have addressed severe oral mucositis remission time, some of them have emphasized the onset time of this condition [4,13]. In addition, the survival analysis technique was used to prepare a regression model to explain the duration of severe oral mucositis and its relationship with a few variables. 

Metastasis affected severe oral mucositis remission time. Patients with metastasis had a shorter duration of severe oral mucositis than non-metastatic individuals. Patients with metastasis are generally subjected to surgical treatment [19,20], and the chemotherapy used in the treatment regimen for the metastasis of some tumors produced high levels of toxicity and responses that were not sufficiently effective [21]. 

MXT is one of most cytotoxic agents used on chemotherapy and is often used on pediatric patients with hematological [22] and solid neoplasms [23] including different phases of treatment. In our study, we reported MXT therapeutic is more associated with non-metastatic and older patients despite length of treatment. This could explain why metastatic and younger patients showed shorter clinical presentations of oral severe mucositis compared with non-metastatic and older groups as shown on final log-normal regression model. 

We could also suppose that patients diagnosed with metastasis require greater care from the healthcare team due to the aggravation of their condition; according to the results, oral care needed to be intensified in patients with metastatic disease. Daily oral monitoring and treatment to resolve oral mucositis were required to help reduce the duration of severe oral mucositis and, consequently, the prognosis of the course of cancer treatment. 

The therapeutic regimen for metastasis requires the use of broader spectrum drugs and/or higher dosage. These factors affect the onset of oral mucositis and its progression to a more severe stage [24]. Delay in the remission of oral mucositis negatively affects the patient’s quality of life to such an extent that it is imperative to interrupt chemotherapy until normal oral mucosal conditions are restored. A reduction in chemotherapy dosage may also be attempted, but it could decrease the likelihood of a cure for the patient. Such an eventuality was reported by Felgenhauer et al. [24] in the evaluation of a group of 24 pediatric patients with metastatic sarcomas, who presented oral mucositis as the non-hematopoietic lesion most frequently related to chemotherapy regimen toxicity. 

Furthermore, cytoprotective agents could also explain the shorter duration of severe oral mucositis Amifostine is a cytoprotective agent which can reduce the intensity of cardiac, hepatic and pulmonary toxicity. It has also been shown to reduce the incidence and severity of oral mucositis [25]. In addition, other cytoprotective drugs have been proven to reduce the onset and duration of severe oral mucositis [13,26]. In another study, it was found that methotrexate-induced cell injury was healed by amifostine, a hydroxyl radical scavenger [27]. Therefore, we reasoned that the duration of severe oral mucositis was shorter in metastatic patients because the chemotherapy protocols they underwent included stomatotoxic drugs. Because of the toxicological nature of these drugs, patients are commonly treated with cytoprotectives, which can affect the tissue repair of the oral mucosa.

The other variable that affected the remission of severe oral mucositis was age. Children aged 10 years or younger exhibited a shorter duration of severe oral mucositis than older patients. 

Younger cancer patients are more susceptible to the onset of chemotherapy-related oral abnormalities [3]. The same study reports that mucositis was the most reported lesion in analyzed records in the 0 to 10 year old age range, demonstrating that oral side effects in children below the age of 12 increased more than two-fold when compared to adult patients. The increased mitotic index of the mucosal cells in the mouth in this group is likely an adjuvant factor.

The cell replication condition, which is greater in childhood, can promote the remission of mucositis, as the damaged cells are quickly replaced by healthy ones. Due to rapid cell division, younger patients develop more severe oral mucositis than older patients when subjected to the same chemotherapy treatment, but the same condition favors the recovery of the younger patients in a shorter amount of time [8,9].

The knowledge about the interference of the factors “metastasis” and “age” in the course of severe oral mucositis is relevant. It drives health professionals to get involved in the care of pediatric cancer patients more attentively in patients that presented such factors, assisting and guiding the decision-making process [28]. Thus, more effective results can be obtained in the control of severe oral mucositis, improving the prognosis and quality of life of these individuals. 

The mean regression time of mucositis was 30.6 days. One study showed an oral mucositis duration of three weeks [15], similar to the time found in this study. Villa and Sonis [13] reported mucosal ulceration between the second and third weeks of treatment. This condition can become contiguous and very painful, may limit function and persist for over a period of two to four weeks after the last radiotherapy dosing. The dosage, type and duration of treatment also affected the duration of mucositis. Alkylating agents and antimetabolite agents, the class most commonly used in the studied sample, had greater mucotoxicity than other chemotherapeutic drugs. Furthermore, bolus infusions were more toxic than other preparations [29]. 

The other results revealed that the majority of patients were males. According to the Brazilian National Cancer Institute (INCA) [30], childhood cancer has a higher incidence in boys than in girls, with the most common tumors being leukemias (affecting white blood cells), those of the central nervous system, and lymphomas (affecting the lymphatic system). Our sample mean age of 7 years was also similar to that of other studies, such as the study performed by Camargo et al. [31], which showed a mean sample age of 6.6. In terms of the skin color variable, most individuals were mixed race, which differs from other studies that have reported that cancer affects white children more often [11,32]. According to the Brazilian Institute of Geography and Statistics (IBGE), the population of Paraíba has a greater number of mixed-race individuals, which may explain the prevalence of such skin color in the study sample [33]. 

A considerable number of patients had a solid tumor diagnosis, which differs from another study that found leukemia to be the most common cancer among children younger than 14 years, especially acute myeloid leukemia, which corresponded to 20% of leukemias among pediatric patients [11]. The incidence of central nervous system tumors increased progressively, which may explain the difference in the sample. Moreover, João Pessoa was one of the capitals that recorded higher bone tumor incidence rates, corresponding to 23.08 per million in habitants [34]. 

The most common treatment regimen was chemotherapy. Approximately 70% of patients suffering from cancer receive antineoplastic chemotherapy during treatment, either in isolation or combined with surgery and/or radiotherapy, the main treatment resource used in children with cancer [10,32]. Chemotherapy may be considered directly toxic to the oral region, as it acts by killing cells with high mitotic activity, such as the ones that compose the oral mucosa [35]. A previous study reported a high incidence of oral mucositis in pediatric patients undergoing chemotherapy, 41% [14]. In our study, all sample patients exhibited severe oral mucositis during follow-up (*n* = 73). 

The results presented provide new information that can contribute to the development of models to explain the duration of severe oral mucositis, providing a breakthrough in knowledge of this condition and enabling the development of more effective strategies for the control of severe oral mucositis.

Nevertheless, some limitations of the present study should be considered. For example, other variables that could influence the duration of severe oral mucositis, such as the patient’s oral status, the dosage of the administered chemotherapeutic agents; hematological and genetic variables were not included. Moreover, the solid and hematological tumors were studied in a single group, which differs from other studies presenting particular characteristics in the occurrence of oral mucositis based on the type of tumor. Despite these differences, the treatment regimens used for Acute Lymphoid Leukemia and Osteosarcoma, the most frequent solid and hematological tumors in our study, respectively, were MXT and Doxorubicin [36,37].

Lastly, there are no reports in the literature of studies investigating the association between the explanatory variables included in our study and the outcome “duration time of severe oral mucositis”, which makes it difficult to compare the findings.

## 5. Conclusions

Understanding the factors that influence the duration of severe oral mucositis may guide the healthcare team to develop more effective strategies to manage pediatric oncological patients presenting with this oral condition. Hence, the findings of the present study point to a greater monitoring and follow-up of patients, particularly the aged ones and those who do not present metastasis, as these conditions prolong the duration of severe oral mucositis.

## Figures and Tables

**Table 1:** Descriptive analysis of oncopediatric patients with severe oral mucositis in Napoleão Laureano Hospital. João Pessoa, Paraíba State, Brazil (2013–2015).

Variable	n	%
Sex		
Male	38	52.1
Female	35	47.9
Age		
Aged ≤ 10 years (1)	47	64.4
Aged > 10 years (0)	26	35.6
Skin color		
Mixed-race	36	49.3
White	22	30.1
Black	14	19.2
Indigenous	1	1.4
Tumor type		
Non-hematological	38	52.1
Osteosarcoma	14	19.2
Willms tumor	13	17.8
Neuroblastoma	3	4.1
Embryonic rhabdomyosarcoma	3	4.1
Germ cell tumor	2	2.7
Adenocarcinoma	2	2.7
Melanoma	1	1.4
Hematological	35	47.9
Acute lymphoid leukemia	26	35.7
Non-Hodgkin’s Lymphoma	6	8.2
Acute Myeloid Leukemia	2	2.7
Hodgkin’s lymphoma	1	1.4
Treatment		
Chemotherapy	46	63.0
Chemotherapy and surgery	19	26.0
Chemotherapy + radiotherapy + surgery	5	6.9
Chemotherapy and radiotherapy	3	4.1
Presence of metastasis		
No	63	86.3
Yes	10	13.7
Bone marrow transplant		
No	71	97.3
Yes	2	2.7
Amputation		
No	47	64.4
Yes	26	35.6
Death		
No	67	91.8
Yes	6	8.2
Chemotherapeutic class *		
Alkylating	16	21.9
Antimetabolite	45	61.6
Natural products	41	56.2
Miscellaneous	8	11.0

* Patients used more than one chemotherapeutic class.

**Table 2:** Log-rank and Peto tests for the study variables of oncopediatric patients with severe oral mucositis in Napoleão Laureano Hospital. João Pessoa, Paraíba State, Brazil (2013–2017).

Variable	Log-Rank	Peto
Sex	0.680	0.470
Age	0.113	0.135
Skin color	0.273	0.553
Hematological tumor	0.426	0.825
Treatment	0.582	0.98
BMT	0.75	0.368
Amputation	0.542	0.74
Death	0.705	0.832
Class 1—alkylating	0.225	0.661
Class 2—antimetabolite	0.103	0.522
Class 3—natural products	0.141	0.432
Class 4—miscellaneous	0.317	1
Metastasis	0.0102 *	0.0337 *

* *p* < 0.10.

**Table 3:** Log-normal regression model with all study variables and considering only significant variables of oncopediatric patients with severe oral mucositis in Napoleão Laureano Hospital. João Pessoa, Paraíba State, Brazil (2013–2017).

**Initial Log-Normal Model with All Variables**		
**Variable**	**Standard Deviation**	***p*** **-Value**
Age	0.2119	0.151
Sex	0.1904	0.732
Death	0.3922	0.548
Skin color	0.2021	0.424
Treatment	0.2697	0.269
Amputation	0.2662	0.534
Metastasis	0.2896	5.81 × 10^−3^
Hematologic tumor	0.2979	0.307
BMT	0.7013	0.490
Alkylating	0.2592	0.290
Antimetabolite	0.4170	0.175
Natural products	0.3715	6.29 × 10^−2^
Miscellaneous	0.3247	0.906
**Final Log-Normal Model**		
**Variable**	**Standard Deviation**	***p*** **-Value**
Age	0.1879	9.16 × 10^−2^
Metastasis	0.2609	4.64 × 10^−2^

**Table 4:** Estimated parameters of the final log-normal regression model fitted to the severe oral mucositis data in oncopediatric patients in Napoleão Laureano Hospital. João Pessoa, Paraiba State, Brazil (2013–2015).

Co-Variable	Estimate	S.E.	*p*-Value	Estimated Coefficients
Constant	2.864	0.1185	6.02 × 10^−129^	exp(2.864) = 17.53151
Age	0.317	0.1879	9.16 × 10^−2^	exp(0.317) = 1.373003
Metastasis	−0.520	0.2609	4.64 × 10^−2^	exp(−0.520) = 0.5945205
Log (scale)	−0.267	0.0843	1.51 × 10^−3^	exp(−0.267) = 0.7656731

**Table 5:** Rate* of Methotrexate uses according to age strata and presence of metastasis with severe oral mucositis in Napoleão Laureano Hospital. João Pessoa, Paraíba State, Brazil (2013–2015).

Variable	MXT Uses during 4 Weeks		MXT Uses during 10 Weeks	
	*n* *	%	*n* *	%
Age				
Aged ≤ 10 years (1)	28	14.9	78	16.6
Aged >10 years (0)	37	35.6	99	37.9
Presence of metastasis				
Yes	6	15.0	19	19.0
No	59	23.4	157	24.9

(*) Superior crude numbers of uses considering multiple uses of MXT despite of times of applications. These numbers could be referred to same or different patients.
